# Ecological Niche Modelling Approaches: Challenges and Applications in Vector-Borne Diseases

**DOI:** 10.3390/tropicalmed8040187

**Published:** 2023-03-25

**Authors:** Pablo Fernando Cuervo, Patricio Artigas, Jacob Lorenzo-Morales, María Dolores Bargues, Santiago Mas-Coma

**Affiliations:** 1 Departamento de Parasitologia, Facultad de Farmacia, Universidad de Valencia, Av. Vicent Andres Estelles s/n, 46100 Burjassot, Valencia, Spain; 2 CIBER de Enfermedades Infecciosas (CIBERINFEC), Instituto de Salud Carlos IIII, C/Monforte de Lemos 3-5. Pabellón 11, Planta 0, 28029 Madrid, Madrid, Spain; 3 Instituto Universitario de Enfermedades Tropicales y Salud Pública de Canarias, Universidad de La Laguna, Av. Astrofísico Fco. Sánchez s/n, 38203 La Laguna, Canary Islands, Spain

**Keywords:** ecological niche models, species distribution models, neglected tropical diseases, mosquito-borne diseases, tick-borne diseases, climate change, global change

## Abstract

Vector-borne diseases (VBDs) pose a major threat to human and animal health, with more than 80% of the global population being at risk of acquiring at least one major VBD. Being profoundly affected by the ongoing climate change and anthropogenic disturbances, modelling approaches become an essential tool to assess and compare multiple scenarios (past, present and future), and further the geographic risk of transmission of VBDs. Ecological niche modelling (ENM) is rapidly becoming the gold-standard method for this task. The purpose of this overview is to provide an insight of the use of ENM to assess the geographic risk of transmission of VBDs. We have summarised some fundamental concepts and common approaches to ENM of VBDS, and then focused with a critical view on a number of crucial issues which are often disregarded when modelling the niches of VBDs. Furthermore, we have briefly presented what we consider the most relevant uses of ENM when dealing with VBDs. Niche modelling of VBDs is far from being simple, and there is still a long way to improve. Therefore, this overview is expected to be a useful benchmark for niche modelling of VBDs in future research.

## 1. Introduction

Diseases transmitted by blood-sucking arthropods, considered as vector-borne diseases (VBDs), pose a major threat to human and animal health since the beginning of time [1]. This kind of diseases accounts for around the 17% of all communicable diseases, causing more than 700,000 human deaths every year [2]. For instance, in 2017 malaria affected an estimated 209 million people globally and caused near 620,000 deaths, while 40,500 deaths were attributed to dengue and an estimated 105 million people suffered the disease in over 129 countries [3,4]. The burden of VBDs is greater in tropical and subtropical areas, with disproportionally higher morbidity and mortality rates among poorer populations [2]. Concerning its economic impact, VBDs represent an immense toll in economies, restricting both rural and urban development [2], e.g., the estimated global cost for malaria was US$ 2.9 billion in 2015 [2], and US$ 8.9 billion for dengue in 2013 [5]. Furthermore, more than 80% of the global population is at risk of acquiring at least one major VBD [2].

The ongoing worldwide environmental changes resulting from human activities have a profound impact on the epidemiology of VBDs [6]. There is plenty of evidence indicating that anthropogenic changes have already altered the transmission of VBDs in a number of ways [7,8,9,10,11]. Besides human-caused disturbances of landscape and an increased connectivity between human populations, diseases transmitted by invertebrate vectors are particularly susceptible to the effects of climate change [12,13]. Indeed, a climate-driven phenomenon of outbreaks of VBDs is occurring worldwide, as recently evidenced during severe El Niño–Southern Oscillation events [14,15,16]. Climate change will affect the distribution of vectors and hence the range over which diseases are transmitted, as well as the efficiency with which vectors transmit pathogens [13,17]. For example, *Aedes*-borne viruses—especially Dengue, Chikungunya and Zika—have shown a rapid geographical spread and an increasing disease burden [18,19], largely attributed to the rapid expansion of its *Aedes* vectors, which, in turn, is associated with climate change and globalization [20,21,22]. Furthermore, climate change can facilitate the seasonal spread of VBDs by widening the yearly transmission window, allowing for local transmission in new areas and increasing the replication rate of pathogens in the vector [12,23]. In this complex scenario, while climate change accelerates and other anthropogenic disturbances increase, modelling approaches become an essential tool to assess and compare multiple scenarios (past, present and future), and further predict the geographic risk of transmission of VBDs.

A model is a simplified representation of a complex system [24], with the aim of helping to better understand the real world. Then, these models are useful to make predictions about the dynamics of the real system. A number of modelling strategies have been applied to foresee the geographic occurrence or transmission of VBDs. Some simpler approaches use the form of early phenological models, nowadays referred as *growing-degree-day* (GDD), to predict the development and availability of invertebrate vectors and/or parasitic infective stages [25]. The GDD is defined as the amount of heat an organism must accumulate to achieve each developmental milestone or to reach full development [25,26,27]. The calculation of this index relies on temperature above a defined threshold and is frequently complemented with other environmental data, such as rainfall and evapotranspiration [28]. Typically, its output is a numerical value, which confers a higher risk of infection at higher values. Its usefulness has been proven in a number of situations, as with ticks [29], sand flies [30], mosquitoes [26] and mosquito-borne diseases [28,31,32,33].

The former forecasting systems are empirical in nature and rely on correlations between historical data. In doing so, these approaches do not explicitly capture the myriad of factors that impact on vectors, hosts and pathogens, giving shape to life cycles and patterns of VBD transmission [34,35]. Therefore, they are argued to have limited ability to assess risk and guide interventions under changing conditions [36]. In turn, mechanistic mathematical models should presumably overcome these limitations. The flexible approach of these models directly incorporates fundamental biological mechanisms, integrating environmental and epidemiological processes and enabling predictions for novel combinations of environmental conditions [37,38,39]. Yet, in essence, these are complex theoretical models and therefore require validation with empirical data [40]. Further, their parameterization and application are costly and time-consuming [41,42], depending on the availability of high-quality environmental and epidemiological data as input (for example, [34,43]), which is usually absent for the majority of species involved and in most of the countries affected by VBDs. In addition, this kind of models might be vulnerable to large-scale effects of seemingly minor assumptions about parameter values [42]. Hence, their usefulness at the broader scales required to guide public health interventions is doubtful and remains to be tested [42].

**Figure 1:**
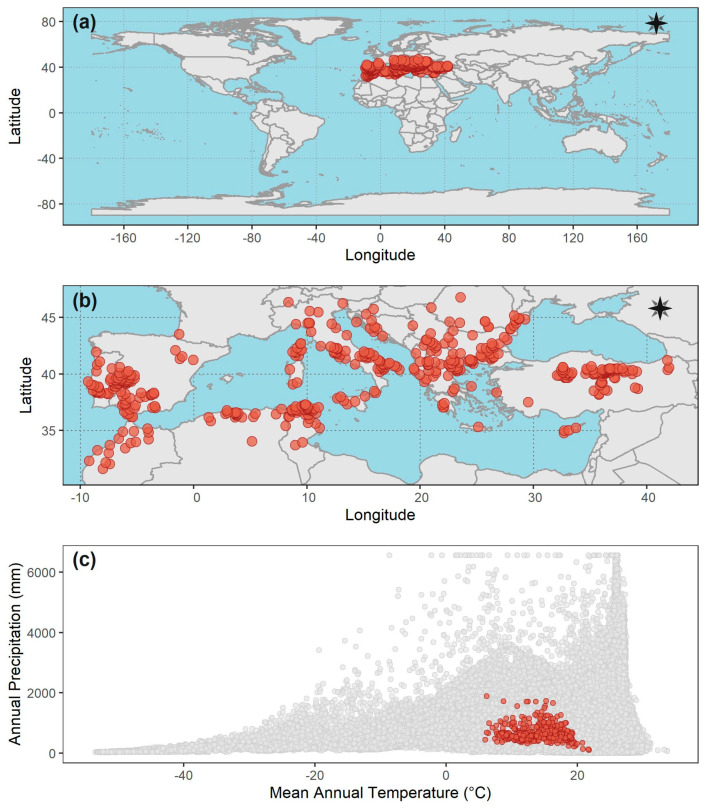
Representation of the Hutchinson’s duality between the geographical and environmental spaces occupied by *Hyalomma marginatum* tick, the main vector of the Crimea-Congo virus [48] (occurrence records were downloaded from the VectorMap data portal [http://www.vectormap.si.edu], accessed on 24 February 2023, and https://doi.org/10.5061/dryad.2h3f2, accessed on 24 February 2023). Worldwide (**a**) and detailed (**b**) geographical distribution of the *H. marginatum* tick (red dots); (**c**) distribution in the environmental space (as defined by mean annual temperature and annual precipitation) of the environmental combinations available worldwide (grey dots, 100,000 random locations chosen worldwide), and the environmental space occupied by the *H. marginatum* tick (red dots, 475 non-duplicated georeferenced records).

A more integrative strategy, ecological niche modelling (ENM) is rapidly becoming the gold-standard method for disease risk mapping [44]. ENM considers known occurrences of species (either parasite, vectors, or hosts) in geographic space, and then infers some form of distribution in environmental space [45]. The result is a picture of the species’ ecological niche, which can be represented and thoroughly analysed in the environmental space, and later projected onto geography to identify a potential distribution for the species (see the Hutchinson’s duality, which states that the local values of *n* environmental attributes of each pair of geographical coordinates in the physical world can be represented in an *n*-dimensional environmental space, delimiting its corresponding niche space and allowing reciprocal projections between geographic and environmental spaces [46], represented in Figure 1). Hence, the distribution of disease occurrences can be seen as the geographic projection of the ecological distribution of the pathogen as constrained by the ecological and geographic potential of each of its interacting species: reservoirs, vectors, etc. [45]. To date, ENM techniques have been used numerous times to model the distribution of vector-borne pathogens or their vector species, i.e., a non-exhaustive search of the combined terms “*niche model* AND *vector*” and “*species distribution model* AND *vector*” (although not equivalent, the terms niche model and species distribution model are frequently used as synonyms—[47]) in the Scopus and Web of Science databases yielded 187 and 226, and 106 and 90 published articles, respectively, in the last two decades.

The purpose of this overview is to provide an insight of the use of ENM to assess the geographic risk of transmission of VBDs, by focusing on a number of crucial issues which are often disregarded when dealing with ENM of VBDs. As proof of concept, we use studies from the three-year period from 2021 to 2023 as listed in Scopus, and, when necessary, relevant literature from other time periods. The reader should also consider the vast literature available on this subject (i.e., [39,49,50,51,52,53,54]).

## 2. Fundamentals of ENM for VBDs

Ecological niche modelling (ENM) provides a basis for understanding the complexities of distributions of species in both geographic space and ecological dimensions [55]. In ecological niche theory, the fundamental niche (**N***_F_*) represents the set of abiotic environmental conditions necessary for the long-term persistence of the species population [24]. However, all the environmental conditions in **N***_F_* may not be entirely available for the species, as it might be constrained by dispersal limitations, biotic interactions, and the evolutionary capacity of the species to adapt to new conditions. This heuristic configuration, named as “BAM” *sensu* Soberon and Peterson [56], centres on requirements of particular abiotic conditions (“A” for abiotic conditions) and how they relate to biotic interactions (“B” for biotic conditions) [56,57]. Finally, the species may be limited from occupying the entirety of its distributional potential by accessibility considerations (“M” for mobility) [56,57]. As such, the conjunction A∩B can be seen as the potential distribution of the species, while A∩B∩M would be a hypothesis of the actual distribution of the species and the current conditions present across it. This portion of the **N***_F_* that is actually occupied by the species is referred as the realized niche, **N***_R_* [24].

Since VBDs (and disease transmission systems in general) are largely the product of interactions among species (pathogens, vectors and hosts), their ecological and distributional dynamics differ from those of more “normal” species. A quite novel framework to ENM of disease systems proposes to treat them from the perspective of disease biogeography (further details in [45]). This framework not only emphasizes parasite–host interactions (“B”) as essential to the process, but requires considering a crucial and formal hypothesis about the area that have been accessible to the species via dispersal over relevant periods of time (“M”) [45,58,59]. A number of issues differentiates the transmission of VBDs from this general framework proposal for disease systems: increased complexity in the web of interactions among species (pathogen–vector–host), at least one obligate interaction (pathogen–vector), in many cases multiplicity of competent vectors and susceptible hosts, and abiotic conditions of great importance in shaping the distribution of climate-sensitive vectors.

## 3. Common Approaches to ENM of VBDs

Ecological niche modelling (ENM) is categorized depending on how explicitly biological processes (e.g., metabolism) are incorporated [60], and can take two main forms: process-based modelling and empirical reconstructions [61]. *Process-based models* (also known as mechanistic or biological) are based on detailed physiological information on the species that make up the system, thus estimating how habitat suitability for the species changes with the environment [40,61]. As aforementioned, parameterizing process-based models requires full knowledge of the factors influencing the distributions of the species involved [40,61]. Despite its inherent complexity, niche models based on ecological processes do not fully capture dispersal and biotic interactions, thus describing something closer to the fundamental niche than the realized niche (actual transmission) [40,62].

In contrast, *empirical reconstructions* (also known as correlative, statistical, or pattern matching models [40]) are based on associations between known geographic occurrences of species and the ecological characteristics of the landscapes in which they occur [61]. They differ from process-based models in that they do not assume known functional relationships between vital rates and environmental variables, allowing a wider range of environmental variables [40]. A major weakness of this approach is that it can be biased by sampling and the influence of other species not included in the study [61]. Since the appearance of “Maxent” [63], a popular modelling algorithm based on a maximum entropy algorithm, correlative models have gained terrain and are by far the most widely used [64].

Correlative methods can be classified into three categories depending on the type of species’ occurrence data used: presence–absence, presence–background (or profile), and presence-only methods (reviewed in [24,60]). Briefly, *presence–absence models* compare environmental conditions where the organism occurs (i.e., presence) vs. where it did not occur upon observation (i.e., absence), providing the probability of finding the species at each place [65]. Since absence data are largely questionable in quality and of limited availability ([24]; and discussed in [55]), a common approach is to simulate these data by generating random points across the study area and to use it in *presence–background models*. This kind of models compare the available environmental conditions in the study area (i.e., background) with the conditions used by the species (i.e., occurrences), providing an index of habitat suitability [60]. At last, *presence-only models* rely solely on the environmental values inferred from the species occurrence in the study area. Modelling algorithms for each category have been listed and reviewed elsewhere [24,60].

Furthermore, the assessment of the geographic risk of VBDs is generally approached from two alternative methodologies, either modelling components (*component-based* approach) or outcomes (*black-box* approach) [66]:

- The *black-box approach* integrates across the entire transmission system, assuming that the final distribution of disease cases summarizes all biotic interactions involved in the transmission [44,61]. This approach is useful when transmission dynamics are poorly understood and data are limited [44,66], as happens for many VBDs (e.g., [67,68,69]). However, the black-box oversimplification in such intricate transmission systems may be perilous, as it neglects the ecological requirements of the individual component species [55,66].
- The *component-based approach* parses the overall transmission cycle into the ecological niches of the individual component species (e.g., pathogens, vectors, hosts) [55]. This approach may allow to distinguish different reasons for presence or absence of disease transmission in an area (i.e., presence/absence of a competent vector and/or susceptible host), but requires potentially lacking in-depth knowledge of the disease system (e.g., the identity and ecologies of relevant species, transmission cycle) [55]. The latter is particularly true for diseases in which the competent vectors might be multiple or poorly known (for instance, see Celone et al. [70], for an approach with the mosquito *Haemagogus janthinomys*, vector of the Mayaro virus).

Choosing between these two approaches for disease distribution modelling should be carried out in accordance with the research question, data availability and implicit assumptions [44,66].

## 4. Relevant Aspects to Consider When Modelling the Niches of VBDs

### 4.1. Accurate Identification of Occurrence Records

Much has been written regarding data sources (occurrence records and environmental data), its quality and concerns. It is not our purpose to make a review on this matter, so only a few relevant concepts will be highlighted (you can find further information in [24,55,60,66,71]). Ecological niche modelling relies on occurrence data for model calibration, which must be revised and curated thoroughly to only include valid occurrence records. Indeed, the problematic of niche modelling relying on biodiversity records with poor taxonomy and/or misidentification was already highlighted more than a decade ago [72]. The advised first step should be to thoroughly check the correct taxonomic identification of the species’ records to include in the study. With exceptions, this step might represent a minor issue with regard to vertebrate hosts. However, when dealing with invertebrate vectors and pathogens, things get trickier. In these cases, specific taxonomic identification is often immersed in controversy. For instance, certain mosquito species of sanitary importance are frequently grouped in complexes of closely related taxa, as their morphology is barely indistinguishable, as the *Culex pipiens* complex [73] or the *Anopheles gambiae* complex [74]. Cryptic species usually have different ecology and host preferences [75], posing an undeniable impact on disease epidemiology and hence modelling procedures. Similarly, the classification of the triatomine kissing bugs transmitting Chagas disease is subdued to constant changes [76,77], which sometimes are related with ecological characteristics (i.e., [78]), as well as occurs with the *Lutzomyia* sand flies transmitting leishmaniasis [79]. Thus, whenever possible, the databases to be used must be filtered and reinforced with a molecular identification (i.e., [69,80,81,82]). Despite the aforementioned, the issue is usually neglected, and most of the studies rely solely on morphological identification (e.g., [83,84,85,86,87,88,89,90,91,92,93,94,95,96], but see [97] for a mixed approach), or do not even describe the identification method [70,82,98,99,100,101,102,103,104,105,106,107,108].

In the case of diseases, occurrence data are represented as disease cases, or serology or direct detection of pathogens or parasites [24,71]. These disease data should have a trustworthy metadata, including traceable diagnostic methods, data sources, transparent surveillance protocols, temporal details and quantified uncertainty (e.g., spatially error, sensitivity of the diagnostic method) [24]. Moreover, critical care must be taken to include only those cases that were infected by ways that are relevant to the models being developed [66], i.e., only those cases with vectorial transmission should be included if the models are to be meaningful estimates of the ecological niche of the disease in question.

### 4.2. Global versus Local Occurrence Data

**Figure 2:**
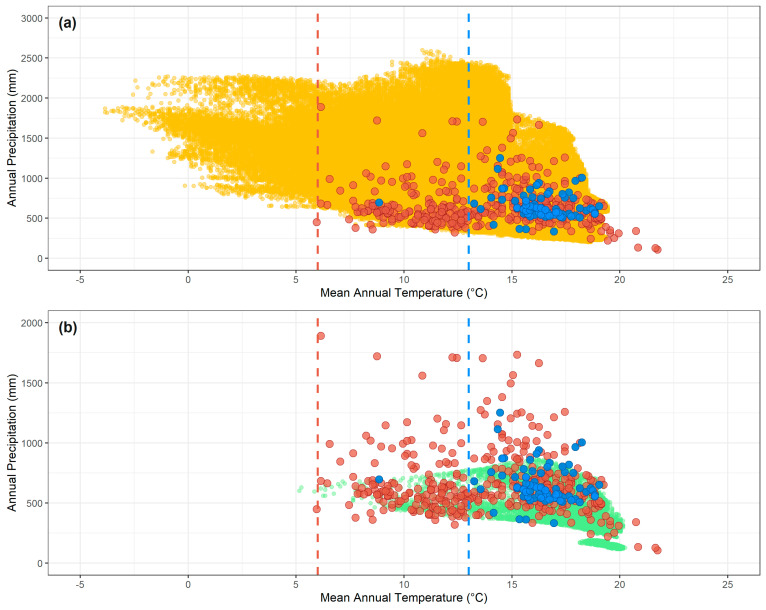
Representation of the environmental spaces available in (**a**) continental Spain (yellow dots), and in (**b**) the Canary Islands (light green dots) as defined by mean annual temperature and annual precipitation. The blue dots represent the environmental space occupied by the *Hyalomma marginatum* tick as restricted to the boundaries of continental Spain (77 non-duplicated occurrence records), whereas the red dots represent the environmental space occupied by its global occurrences (475 non-duplicated occurrence records). (**a**) A niche model of the *H. marginatum* tick, based solely on the occurrences in continental Spain (blue dots), will truncate the lower limit of the species’ thermal tolerance. Most of these boundary-restricted occurrences (blue dots) have been recorded in areas with mean annual temperatures superior to 13 °C (blue dashed line), while the species withstands mean annual temperatures as lower as 6 °C (red dots, red dashed line). Thus, a projected distribution based on this truncated niche model will underestimate those environments available in Spain with mean annual temperatures between 6 °C and 13 °C (between red and blue dashed lines). (**b**) A similar situation will occur if this same truncated niche model should be transferred to the Canary Islands to identify areas suitable for the establishment of *H. marginatum* populations. Such a model will fail to predict those areas in Canary Islands presenting mean annual temperatures between 6 °C and 13 °C (between red and blue dashed lines).

A seldom treated, but crucial issue in ENM of VBDs, concerns the geographical extent of the occurrence data. Despite being acknowledged long ago in ecology [109,110]), fitting ENM with focus only on a part of the entire distribution of a species continues being a common approach [111]. As far as we know, this is what occurs with a large amount of the studies dealing with VBDs. As these kinds of studies are usually performed with public health purposes at regional or local scales, they often rely on regional/national datasets collected by administrations or NGOs whose range of action is defined by restricted geographical or political borders (i.e., country or even provinces’ administrations) (e.g., [67,68,69,80,83,84,85,86,87,88,89,90,91,92,93,94,95,96,98,99,101,104,105,106,107,108,112,113,114,115,116,117]). The concern resides in the fact that ENM built with occurrence data restricted to artificial boundaries might consider only a subset of the environmental conditions experienced by a species across its entire range (i.e., “spatial niche truncation” [118]); therefore, providing an incomplete description of the environmental limits [109] and underestimating the environmental conditions that the species can withstand [111] (see an example in Figure 2).

Spatial niche truncation is particularly problematic when the aim is to project the prediction to other areas or time-periods not found in the calibration area (that is to say, to extrapolate) [118]. Conversely, it has been argued that partial (truncated) datasets may be better to identify other distribution constraints, which may differ in different parts of the distribution range [60]. Such is the argument alleged by Tjaden et al. [119] when proposing a “non-tropical” ENM to assess the transmission of the chikungunya virus outside the tropics. According to these authors, the ENM restricted to non-tropical regions predicts chikungunya transmission in Europe with greater accuracy than the previous global ENMs that were based on predominantly tropical occurrence locations (e.g., [119,120]). In any case, if not appropriately justified (e.g., [119,121]), the prudent approach is to use global over local data in spite of the area of interest (for example, [70,81,82,97,100,102,103,122]; but see [123,124] for a dual approach). Otherwise, models built upon partial datasets risk providing a biased description of the species’ niche [109,125], and thus failing to forecast the complete species range [126].

### 4.3. Importance of Defining M for Background Selection

As aforementioned, *presence–background models* (with Maxent the most popular) contrast the environmental characteristics of sites of known occurrence against those associated with background locations where presence/absence is unmeasured [127]. Being this essential to predict the probability of presence, it is crucial to outline the calibration area as the area that has been accessible to the species (**M**) [58,128], as the species will be absent from outside of this area for reasons unrelated to **A** [129] (such as geographical barriers [130] or anthropogenic range contractions [131]). Defining **M** has strong implications in several aspects of ENM, for instance:

(i) Being **M** the geographic extension across which the contrasts should be developed [132], it determines the area within which presences may exist and within which absences are meaningful, in that they represent sites with the broader background landscape actually likely to have been “tested” by the species for suitability, but not occupied [58]. Thus, to minimize the impact of assumptions about absences from areas that are not accessible to the species, the background sample should be chosen to reflect the environmental conditions that one is interested in contrasting against presences [128].
(ii) **M** has effects on model validation, as areas outside **M** (where the species cannot occur, owing to restrictions resulting from **M** but not **A**) will generally be predicted at lower suitability levels. In consequence, inclusion of these areas (which hold no presence data, but often includes absences that are more distant environmentally from the presences) makes the model look better than it actually is [58].

A conscientious estimation of **M** should be carried out prior to initiation of analyses, and for this a number of approaches have been proposed:

- *Geopolitical boundaries*: a frequently applied approach is to use administrative boundaries (e.g., municipality, department, province and country) to delimit the calibration area [24,58]. However, this pragmatical use of geopolitical boundaries without an explicit, a priori hypothesis regarding the extent of **M** presents a major weakness: restricting models based on administrative areas does not account for the biology of the organism [24]. Indeed, most of the cases that applied this approach lacked an explicit assumption about **M** [67,68,80,83,84,85,86,88,89,91,92,93,94,95,98,99,101,104,106,107,108,109,110,112,116]. Since organisms do not know about geopolitical borders, the perils of this common failure will result in models that are misaligned with the ecology of the organism, resulting in underestimations of the true potential of the disease spread [24] (somehow related to the spatial niche truncation mentioned before).
- *Occurrence buffering*: an often-used approach consists of determining an optimal buffer area around the occurrences. The radius buffer can be selected in consideration of the dispersal potential of the species [69,81,87,97,101,105,114,117], or after assessing the performance of a series of test models based on buffer with increasing radii (e.g., [100,119]).
- *Biogeographical units*: these geographical areas are categorized in terms of their biotas [133,134], being defined based on distinct sets of endemic taxa and communities [134]. These areas are usually limited by geographical barriers, altitudinal ranges or a vegetation type [134]. Therefore, the boundaries of the biotic regions within which a species is known to occur may be informative about the barriers that have constrained its distributional potential, resulting in a reasonable hypothesis of the areas that have been available for the species over relevant time periods [58]. To account for potential dispersal beyond these boundaries, an additional buffer can be created around each biogeographical unit occupied [135], or just around the occurrence points within certain distance from the borders of the established biogeographic area [136]. This approach is quite simple, and may prove the most operational [137]. In fact, it has been applied in a number of opportunities to define the accessible area of mosquitoes, kissing bugs and others (i.e., [87,102,117,124,136]).
- *Niche-model reconstructions*: a simple present-day niche model could be used to estimate the basic dimensions of a species’ distributional potential, and then could be back-projected over the historical conditions that the species has experienced (e.g., Pleistocene Last Glacial Maximum, Last Interglacial) [58,66]. These estimates of past distributional areas can then be combined in a proxy of the long-term dispersal potential, providing a broad initial hypothesis of areas that have been accessible to the species (**M**) to be used in a second round of model calibration [58,66]. Despite this approach risks some circularity in its implementation, is operational and could be implemented readily [58]. Furthermore, this risk of circularity, owed to the lack of an explicit hypothesis of **M** in the initial round of modelling [58], could be accounted for by combining the two approaches already described: the biogeographical approach could be used to define **M** in the initial calibration round, to be later back-projected and used to estimate **M** in the second round of modelling.
- *Full dynamic dispersal models*: a more realistic approach should join estimates of the niche with scenarios of dispersal potential through periods of environmental change, considering explicitly the spatially path-dependent nature of effects of environmental change on species’ dispersal reach and consequent distributional potential [58]. Despite its computational challenges, a first akin simulation of this general framework was outlined by Barve et al. [58], and later extended by Machado-Stredel et al. [132]. This intricate approach of defining **M** is based on a cellular automata simulation where dispersers colonize (or died out) in a cell grid that has suitable conditions drawn from a preliminary estimate of the fundamental ecological niche. These processes are replicated several times to generate an account of accessed cells, to be later summarized in an estimate of **M** that houses the most frequently accessed cells. This simulation-based method presents a quantitative approach for estimating the accessible area of a species under biologically realistic assumptions, offering the possibility of incorporating relevant climate changes into this estimation of environmental suitability across space and time [132]. As far as we know, this approach has not been applied yet in the modelling of VBDs.

### 4.4. Sampling Bias and How to Deal with

Sampling bias is pervasive in most datasets of occurrence records, and represents a major concern when developing ENM (detailly explained in [55,128]). Briefly, a disproportionate geographical or environmental sampling will likely hamper the predictive ability of the niche model based on that sampling [55,138]. Inextricably linked to collection methods, the spatial pattern of occurrences is mainly driven by accessibility [139]. Indeed, the majority of records for all terrestrial groups fell within 2.5 km of a road [139]. The sampling bias not only affects the spatial distribution of occurrences from vertebrate hosts, but also from invertebrate vectors (e.g., insects [140]). This is even more relevant when considering the occurrence of pathogens, often recorded as disease reports. For instance, disease data are generally aggregated at administrative levels (e.g., province, country), where the geographic site of infection is usually not reported and is instead referred to the health facility where it was diagnosed [44]. At last, if the bias is not accounted for, the fitted model might be closer to a model of sampling effort than to a model of the true niche of the species [141].

Despite the sampling bias and the consequent non-independence of occurrences needing to be accounted for, a good number of the VBDs studies published in the last three years omitted declaring any procedure for dealing with the sampling bias [67,68,80,81,82,83,84,85,86,88,89,91,94,95,96,98,101,104,108,113,124,142].

The most straightforward approach would be to manipulate the occurrence data [141]. Perhaps not the ideal, because it disregards the distribution of the sampling effort, thinning of occurrence records either in environmental space or geographic space is a viable solution [143]. Geographic and environmental thinning are conceptually equivalent, as they use a distance measure to determine a filter size [144]. Geographical thinning involves two methods [143]: i) overlaying an equal-area grid on the study region and randomly sampling a set number of occurrence records (e.g., one) from each grid cell (Hijmans and Elith, in [143]); or ii) randomly removing occurrence records so that no two are closer than a given linear distance [145,146] (you can find an easy-to-implement method in [143]).

On the other hand, spatial bias usually leads to environmental bias because of the over-representation of certain environmental features of the more accessible and extensively surveyed areas [145]. Moreover, geographical thinning might enhance a disproportionate sampling in the environmental space [147,148]. Therefore, geographical thinning should be used with caution, and the use of an environmental thinning technique is highly recommended [144,147]. For this, Varela et al. [147] proposed to place a regular grid with a specified cell size to a multivariate environmental space, so as to randomly choose one occurrence from each grid cell and remove the rest from further analysis (the method is fully described in [147] and available in https://github.com/SaraVarela/envSample, accessed on 27 February 2023). For an application with VBDs see [90,102].

Like all thinning approaches (both geographical and environmental), the optimal degree of thinning remains subject to empirical determination [143]. However, the main drawback of these manipulations is the resulting discard of a number of occurrence records, which in many cases might be unaffordable [141]. An alternative approach, which avoids the loss of occurrence records, is to manipulate the selection of the background data so that these share the same bias as the occurrence records [149]. This approach stands on the assumption that a similarly biased background nullifies the bias in the occurrence records [141]. One largely used method consists of selecting the background data based on a *target group* [141], which restricts the background to locations with recorded presence of a particular group (usually a higher-rank taxon), including the modelled species. This method assumes that the presence records of the target group reflect the sampling probability distribution that led to the presence records of the modelled species [146] (e.g., [82]). Alternatively, a recently proposed *background thickening* method relies on increasing the density of uninformed background locations around presences [150]. Briefly, this method samples background locations from an estimate of sampling probability based exclusively on density of occurrences [150], emphasizing the comparison between a presence and its surroundings.

In all, it has been suggested that accounting for sampling bias in environmental space is more efficient than doing so in geographical space [151]. However, if many occurrence records are available, geographical filtering and balancing of occurrence data should be preferred relative to background manipulation [145]. Indeed, this method seems to be by far the most frequently used (e.g., [69,70,81,83,87,92,93,97,100,103,105,106,107,112,116,117,119]). However, if only few spatially clustered occurrence records are available, the manipulation of the background dataset seems to be the second-best option [145].

### 4.5. Model Complexity and Fine Tuning of the Model Calibration

Ideal niche models oscillate in a delicate balance between the precision that is possible with highly complex models and the general predictive power that can derive only from sufficiently simple models [152]. Often, model complexity has been considered as unimportant [153]. However, models that are inappropriately complex or inappropriately simple show reduced ability to infer habitat quality, reduced ability to infer the relative importance of variables in constraining species’ distributions, and reduced transferability to other time periods or geographical regions [153]. Indeed, many models have been developed that are overly complex and that lack predictive power [154]. Despite the choice of algorithms or estimation frameworks, model calibration is a key process in which variable selection and model settings are fine-tuned to obtain the best model performance [64,155]. Unfortunately, in spite of the recommendations for maximizing reproducibility of ENMs [64,156], the information concerning model calibration is lacking in a high number of studies dealing with VBDs [80,82,83,84,85,86,88,89,91,92,96,98,107,112,113,124].

Given the resource-demanding and time-consuming process of model calibration, many empirical studies rely on default settings of a given algorithm/software package [64,157] (e.g., [67,68,70,93,94,101,116]). This click-and-run modelling framework is based on the use of “recipes” to model the distribution of any species, neglecting the biology of the organism in question [24], and thus a common practice ought to be avoided. Indeed, the species-specific tuning of model parameters affects model complexity [158] and have been shown to improve the performance of ENM [159]. Hence, in recent years, a number of initiatives have dealt with the automatization of the calibration process in order to increase robustness, performance and reliability of ENMs. Most of these initiatives are meant for Maxent, the most popular software for ENM, in the form of open-source R packages (e.g., *ENMeval* [155] and its updated version *ENMeval 2.0* [160], *wallace* and its updated version *wallace 2* [161,162], *kuenm* [163]). A few additional R packages allow for the fine-tuning of hyperparameters from other algorithms, such as *SDMtune* (artificial neural networks, boosted regression trees, maximum entropy and random forest) [158]. Therefore, testing of optimal parameters settings and model selection approaches have now become the rule in the field [155]. Some examples of its application in VBDs are found in [69,81,87,90,95,97,99,100,102,103,104,105,106,108,114,117,119].

In order to optimize model complexity and performance, not only the optimal parameters settings should be determined, but also the predictors used to build the model should be carefully selected [158]. The number of predictors included in the model determine the specific dimensions of the environmental space to be modelled [55]. A highly dimensional environmental space will yield overly complex, and most probably overfitted, models [55,155]. In addition, highly collinear variables allow alternative model structures to yield very similar model fits [156]. Dealing with multicollinearity could be achieved with a number of strategies (revised in [155]), which will reduce the dimensionality of the environmental space and lead to a reasonable set of predictors [55]. However, a recent analysis advocates for exhaustive searches inspecting all possible combinations of environmental variables, in combination with the different value sets for other parameters of interest [155]. This proposed approach is somehow related to the information-theoretic approach of model selection and multimodel inference from traditional multivariate statistics [152]. Despite its relevance, as far as we know, the selection of environmental predictors is only supported by the R packages *kuenm* [163] and *SDMtune* [158].

### 4.6. Unveiling the Uncertainty Inherent to ENM

Model uncertainty is a consistent, but largely unassessed, concern in ENM [152]. Although it is mainly due to the many assumptions underlying the modelling approach [164], this is not the only cause. There are also other sources, such as the uncertainty inherent to the incoming data and their scale, the modelling algorithm and its parameterization, and the chosen climate models when dealing with future projections (general circulation models and representative concentration pathways) [42,152,164,165]. Furthermore, the aforementioned approaches to deal with model complexity and calibration allow to use multiple sets of parameters [163], which might result in more than one parametrization giving an adequate model. This use of multiple parameters settings and replicates gives rise to additional sources of variation in model outcomes [152]. Hence, measurement of certainty or uncertainty are crucial when performing rigorous model calibration processes [166].

An additional source of uncertainty that should be accounted for concerns areas of strict extrapolation. This means detecting areas with novel combinations of environments outside those in **M**, which are areas with conditions extending beyond those over which the model was calibrated [59]. Interpretation of predictions in transfer areas of strict extrapolation may lead to erroneous conclusions [59]. When the physiological response of the species in those areas is unknown, caution is advised about assuming that a species could potentially establish populations or not in such environmental conditions [166]. The environmental similarity between calibration and transfer regions should always be quantified prior to model transfer, so that the interpretation of final models can incorporate the levels of uncertainty and extrapolation caused by model transferability [167,168]. For this, we acknowledge at least two different metrics which identify nonanalog conditions between calibration and transfer regions highlighting regions in geographic space where strict extrapolation occurs:

(i) the Multivariate Environmental Similarity Surface (MESS) readily incorporated in the Maxent software [169].
(ii) the Mobility-Oriented Parity (MOP), available in the *kuenm* R package [163], which modifies and extends the MESS approach [59].

The Mobility-Oriented Parity outstands as it reflects more closely the reality of environmental difference from the set of conditions represented across **M** and experienced by the species [59], offering more robust measures of extrapolative conditions [163]. This analysis of nonanalog conditions via extrapolation metrics is beginning to be regularly applied in the ENMs of VBDs (MESS [102,119,170]; MOP [69,81,87,97,103,117]).

Identifying areas with high associated uncertainty in model projections is crucial to giving a clear picture and avoiding misinterpretations of the potential distributions of disease vectors [55,166]. Even more when the primary objective is model transfer [171]. Evincing the uncertainty from various sources and manifested on multiple levels entails certain difficulty [166], but recent advances in hierarchical modelling could offer a solution [171]. Hierarchical partitioning analysis of the uncertainty, available via the *kuenm* R package [163], let error estimates to propagate through various submodels within one ‘integrated statistical pipeline’ [171], allowing to assess magnitudes, sources and patterns of variation from the different sources [166]. To sum up, model projections must be accompanied by a spatially referenced analysis of the uncertainties [164], such as the reader is given a fair idea of the confidence in predictions for any particular species or place [42]. Even though the predictive capacity of ENMs is only applied along with full appreciation of the inherent uncertainty [165], many studies still omit including their uncertainty results (for example, [80,82,84,85,86,88,89,90,91,92,93,94,95,96,98,100,101,104,105,106,107,108,113,116,124,141,172]; but this seems to be changing (e.g., [67,69,70,81,83,97,99,102,103,114,117]).

## 5. Main Applications

Ecological niche models are most often used in one of four ways [153]: (i) to estimate the relative suitability of habitat known to be occupied by the species, (ii) to estimate the relative suitability of habitat in geographic areas not known to be occupied by the species, (iii) to estimate changes in the suitability of habitat over time given a specific scenario for environmental change and (iv) as estimates of the species niche. In the following paragraphs, we briefly present what we consider the most relevant uses of ENM when dealing with VBDs.

### 5.1. Disease Distribution and Risk Mapping

If a comprehensive understanding of the natural history of the disease (pathogen/vector) system is available, a logical use of ENM is for the identification and characterization of distributional areas meeting the ecological requirements for disease transmission [173]. This approach not only aids to predict disease distribution, but also allows to characterise the ecological requirements of the species involved, which may be unknown or poorly described [61]. Depending on the approach (the previously discussed *black box* vs. *component-based* approaches), the modelling process may only include reports of human or animal disease to summarise the entire disease system, or may incorporate occurrence records of vector and hosts to identify the overlapped area among system components. Since the access to trustworthy disease occurrence data are still restricted, the distribution modelling of vector-borne pathogens is limited in either of the approaches (*black-box approach* [67,68,69,98,112,119,172]; *component-based approach* [81,85,87,101]). As a matter of fact, the most frequent is the modelling of the various vector species involved in the transmission of VBDs (e.g., [70,83,84,86,88,89,90,91,92,96,99,102,103,104,105,106,107,114,115]). Even though still not perfect, the distribution modelling of either of the components of a VBD improves the identification of potentially risky areas suitable for transmission, allowing for disease control and planning in the form of informed interventions and appliance of control/palliative measures.

### 5.2. Filling Gaps in Transmission Cycles

As mentioned before, disease transmission can be conceptualized as the integration of the various ecological niches and distributions of each of the species participating in the transmission cycle [45]. Therefore, the characterization of distributional areas via ENM can be applied to identify unknown elements of the transmission cycle, such as vectors or hosts [173]. The geographical distribution of disease occurrences can provide an indication of which suspected taxa are potentially involved and which are not [173], i.e., when one of those species is unknown, ENM can be used in an exploratory sense to narrow possibilities and hypothesize which species may be involved [55]. An exemplary application of this approach can be found in Lippi et al. [116], where the authors analysed the contribution of the tick *Dermacentor variabilis* in the transmission of a pathogenic *Rickettsia* species from the spotted fever group (i.e., *R. montanensis*). Another good example is the one provided by Cuervo et al. [81], who analysed the geographical distribution of the West Nile virus in Spain, and could attribute the higher risk of transmission in southern Spain to the presence and abundance of the mosquito species *Culex perexiguus*.

### 5.3. Assessing the (Potential) Distribution of Invasive Species

An invasive species is defined as a species able to colonize geographical areas beyond their native distributional range, establishing viable populations [174]. During the last century, this extra-range dispersal of species has dramatically increased due to human-mediated processes (i.e., globalisation) [175]. The transmission of VBDs is not excluded from this phenomenon, as disease vectors and pathogens are spreading across continents due to human transport, land-use change and climate change [176]. The use of ENM can help to assess and foresee the geographical extent of these (potential) invasions by disease vectors and vector-borne pathogens [173]. Appropriately and correctly calibrated ENM can be transfer beyond the boundaries of the species’ native range, seeking for environmental conditions that suite the ecological requirements for a species, even if the species is not present there [173,177]. However, when areas accessible to the species across its native distribution do not represent the full spectrum of environmental conditions that the species can tolerate (i.e., a truncated niche), the potentially invaded area is often underestimated [177,178]. To overcome this issue, the use of integrated data on closely related species (at a supraspecific level) allows a more complete characterization of fundamental niches, improving the predictive ability of potentially invaded areas [178]. Not surprisingly, the majority of studies dealing with invasive disease vectors refer to mosquito species, mainly of the genera *Aedes* and *Anopheles*. For instance, Adeleke et al. [100] found that wind speed limits the invaded area by *Aedes albopictus* in Europe, whereas Echeverry-Cardenas et al. [124] and Moo-Llanes et al. [117] analysed the potential geographic distribution of this same species in Colombia and Mexico, respectively. Finally, Valderrama et al. [108] assessed the potential distribution of *Anopheles pseudopunctipennis* in northern Chile.

### 5.4. Keeping up with Climate and Global Change: Changes in Time

The broad, correlative power of ENM provides a potent tool to address the question of likely geographic shifts in distributional areas of species or phenomena under scenarios of climate change [66,173]. To this aim, ENM appears more suitable than mechanistic models for the vast majority of disease systems, as they do not depend on a transmission model and the assumptions that are required [41,66]. Despite its extended use, many cautions and caveats are necessary when applying ENM for forecasting the future distribution of species as climate change proceeds. This subject is reviewed in detail by Peterson et al. [152], but three aspects that ought to be considered are:

(i) Effects of niche truncation on model transfers to future climate conditions;
(ii) Effects of model selection procedures on future-climate transfers of ecological niche models;
(iii) Overall variance (uncertainty) in model outcomes.

In this sense, we have analysed 12 studies published in the last three years [68,87,94,95,101,105,109,112,115,124,142,170]. From these, only two studies considered the global occurrences of the modelled species [124,142]. Meanwhile, the rest restricted their data to local occurrences, mostly at the national level [68,87,94,95,101,105,109,112,115,170], suggesting that a niche truncation effect might have hampered their future projections. Furthermore, only five of them presented some kind of extrapolation assessment [69,87,112,115,170], but no overall variance of the model outcomes.

A further aspect to consider is the number of climate scenarios to be used in the niche model projections. It has been suggested that to represent a decent amount of uncertainty in climate model projections, a minimum of five non-related models ought to be selected [179]. Future climate projections represent, at least to some degree, a sample of uncertainty of future climate evolution [179]. Therefore, the use of multiple climate scenarios in the niche modelling procedures allows to capture its inherent variance. However, out of the studies analysed, only three of them considered more than one climate model [95,109,115]. Yet, all of them failed to present any uncertainty assessment of their future climate projections, meaning that the confidence of their predictions might be compromised.

### 5.5. Keeping up with Climate and Global Change: Changes in Space

Climate and global change should not be exclusively seen as a driver of geographical shifts in the future, since many native species are already shifting their ranges to keep up with climate change [180]. Invasive species are excluded from this consideration, as in general they have not reached a distributional equilibrium in the invaded range by fulfilling a niche similar to the original [181]. Thus, an additional application of ENM is to assess the availability of suitable habitats which might explain and forecast the (potential) distributional shifts of hosts and vector species. Distributional shifts might involve either range expansions or contractions. Range expansions are driven by the dispersal of individuals away from a population’s core and can be particularly important following a shifting climate envelope, where edge patches may become thermally suitable at different times [182].

Conversely, during a contraction, a species’ range gets fragmented due to climatic heterogeneities, which usually leads to different subpopulations residing in small refuge areas [183]. In this sense, ectothermic and thermophilic species are assumed to benefit from increasing temperatures, giving place to a range expansion at higher latitudes and altitudes [184]. Indeed, a northward expansion is projected in a number of tick species [93,170,184,185] (reviewed in [186]). However, the opposite is also feasible (range contraction), as the upper limits of a species thermal tolerance might be reached due to the continued warming, with negative consequences for thermophilic species (as an example, [187,188,189]). Indeed, a range contraction is expected for *Lutzomyia intermedia* (a tropical sand fly vectorizing *Leishmania braziliensis*), while other subtropical *Lutzomyia* species (*L. neivai*) is predicted to shift its range southwards in southern South America [189].

In summary, climate change will not always lead to range expansion of VBDs [189], and ENMs should be considered thoroughly to foresee the forthcoming changes.

### 5.6. Aiding to Disentangle Species Complexes

Due to the aforementioned complexities of taxonomic identification among numerous taxa of vectors, ENM may be useful for delimiting species and populations [55]. Since morphological differentiation is unreliable in these taxa complexes, identification relies on phenotypical or molecular characters. However, ecological characters can offer additional, complementary information [55,189] (reviewed in [190]), giving the opportunity to identify populations that are differentiated in ecological features [191]. For instance, such an approach has been used to analyse the climatic niches of closely related taxa of ticks [97], kissing bugs [82] and mosquitoes [192], providing an insight on the evolutionary patterns of these problematic taxa.

Furthermore, this approach aids to reach a more complete understanding of vectors’ distribution based on the, often, limited available data [193]. For instance, ENMs provided clues on the ecological preferences of the species involved in the *Anopheles maculipennis* complex [80]. However, if during the diversification of closely related taxa, niche divergence is absent, ENM should rely on all available occurrences unified at the suprataxa level [193]. Such approach was the one followed to model the potential distribution of *Culex pipiens-restuans* complex in Canada [90].

## 6. Concluding Remarks

It is clear that niche modelling of VBDs is far from being simple, and that there is still a long way to improve. However, and despite the difficulties, ENM provides the best available tool for the assessment of diseases geographical distribution and disease risk mapping, as it has been already argued [44]. Indeed, this is what models are: educated guesses about the future [164]. By knowing the underlying assumptions, improving the calibration process, evaluating their potential effects and incorporating the inherent uncertainty in their interpretation, we can narrow the guesses to define the more probable futures. This overview is expected to be a useful benchmark for niche modelling of VBDs in future research.

## Data Availability

The datasets generated for this study are available on request to the corresponding author.

## References

[1] Gubler D. (2009). Vector-borne diseases. Rev. Sci. Tech..

[2] World Health Organization (2017). Global Vector Control Response 2017–2030.

[3] Roberts N.L., Mountjoy-Venning W.C., Anjomshoa M., Banoub J.A.M., Yasin Y.J. (2018). Global, regional, and national incidence, prevalence, and years lived with disability for 354 diseases and injuries for 195 countries and territories, 1990–2017: A systematic analysis for the Global Burden of Disease Study. Lancet.

[4] Roth G.A., Abate D., Abate K.H., Abay S.M., Abbafati C., Abbasi N., Abbastabar H., Abd-Allah F., Abdela J., Abdelalim A. (2018). Global, regional, and national age-sex-specific mortality for 282 causes of death in 195 countries and territories, 1980–2017: A systematic analysis for the Global Burden of Disease Study 2017. Lancet.

[5] Shepard D.S., Undurraga E.A., Halasa Y.A., Stanaway J.D. (2016). The global economic burden of dengue: A systematic analysis. Lancet Infect. Dis..

[6] Sutherst R.W. (2004). Global Change and Human Vulnerability to Vector-Borne Diseases. Clin. Microbiol. Rev..

[7] Afshan K., Fortes-Lima C.A., Artigas P., Valero M.A., Qayyum M., Mas-Coma S. (2014). Impact of climate change and man-made irrigation systems on the transmission risk, long-term trend and seasonality of human and animal fascioliasis in Pakistan. Geospat. Health.

[8] Diuk-Wasser M.A., VanAcker M.C., Fernandez M.P. (2021). Impact of Land Use Changes and Habitat Fragmentation on the Eco-epidemiology of Tick-Borne Diseases. J. Med. Entomol..

[9] Morand S., Lajaunie C. (2021). Outbreaks of Vector-Borne and Zoonotic Diseases Are Associated With Changes in Forest Cover and Oil Palm Expansion at Global Scale. Front. Vet. Sci..

[10] Tamarozzi F., Rodari P., Salas-Coronas J., Bottieau E., Salvador F., Soriano-Pérez M.J., Cabeza-Barrera M.I., Van Esbroeck M., Treviño B., Buonfrate D. (2022). A large case series of travel-related *Mansonella perstans* (vector-borne filarial nematode): A TropNet study in Europe. J. Travel Med..

[11] Wilke A.B., Benelli G., Beier J.C. (2021). Anthropogenic changes and associated impacts on vector-borne diseases. Trends Parasitol..

[12] Caminade C., McIntyre K.M., Jones A.E. (2019). Impact of recent and future climate change on vector-borne diseases. Ann. N. Y. Acad. Sci..

[13] Ogden N.H., Lindsay R. (2016). Effects of Climate and Climate Change on Vectors and Vector-Borne Diseases: Ticks Are Different. Trends Parasitol..

[14] Anyamba A., Chretien J.-P., Britch S.C., Soebiyanto R.P., Small J.L., Jepsen R., Forshey B.M., Sanchez J.L., Smith R.D., Harris R. (2019). Global Disease Outbreaks Associated with the 2015–2016 El Niño Event. Sci. Rep..

[15] Chretien J.-P., Anyamba A., Small J., Britch S., Sanchez J.L., Halbach A.C., Tucker C., Linthicum K.J. (2015). Global Climate Anomalies and Potential Infectious Disease Risks: 2014–2015. PLoS Curr..

[16] Chambaro H.M., Hirose K., Sasaki M., Libanda B., Sinkala Y., Fandamu P., Muleya W., Banda F., Chizimu J., Squarre D. (2022). An unusually long Rift valley fever inter-epizootic period in Zambia: Evidence for enzootic virus circulation and risk for disease outbreak. PLoS Negl. Trop. Dis..

[17] Cavicchioli R., Ripple W.J., Timmis K.N., Azam F., Bakken L.R., Baylis M., Behrenfeld M.J., Boetius A., Boyd P.W., Classen A.T. (2019). Scientists’ warning to humanity: Microorganisms and climate change. Nat. Rev. Microbiol..

[18] Mayer S.V., Tesh R.B., Vasilakis N. (2017). The emergence of arthropod-borne viral diseases: A global prospective on dengue, chikungunya and zika fevers. Acta Trop..

[19] Messina J.P., Brady O.J., Golding N., Kraemer M.U.G., Wint G.R.W., Ray S.E., Pigott D.M., Shearer F.M., Johnson K., Earl L. (2019). The current and future global distribution and population at risk of dengue. Nat. Microbiol..

[20] Kraemer M.U.G., Reiner R.C., Brady O.J., Messina J.P., Gilbert M., Pigott D.M., Yi D., Johnson K., Earl L., Marczak L.B. (2019). Past and future spread of the arbovirus vectors *Aedes aegypti* and *Aedes albopictus*. Nat. Microbiol..

[21] Ryan S.J., Carlson C.J., Mordecai E.A., Johnson L.R. (2019). Global expansion and redistribution of Aedes-borne virus transmission risk with climate change. PLoS Negl. Trop. Dis..

[22] Artigas P., Reguera-Gomez M., Valero M.A., Osca D., Pacheco R.D.S., Rosa-Freitas M.G., Silva-Do-Nascimento T.F., Paredes-Esquivel C., Lucientes J., Mas-Coma S. (2021). Aedes albopictus diversity and relationships in south-western Europe and Brazil by rDNA/mtDNA and phenotypic analyses: ITS-2, a useful marker for spread studies. Parasites Vectors.

[23] Mas-Coma S., Valero M.A., Bargues M.D. (2009). Climate change effects on trematodiases, with emphasis on zoonotic fascioliasis and schistosomiasis. Vet. Parasitol..

[24] Escobar L.E. (2020). Ecological Niche Modeling: An Introduction for Veterinarians and Epidemiologists. Front. Vet. Sci..

[25] Bergquist R., Luvall J.C., Malone J.B. (2021). The changing risk of vector-borne diseases: Global satellite remote sensing and geospatial surveillance at the forefront. Geospat. Health.

[26] Mushegian A.A., Neupane N., Batz Z., Mogi M., Tuno N., Toma T., Miyagi I., Ries L., Armbruster P.A. (2021). Ecological mechanism of climate-mediated selection in a rapidly evolving invasive species. Ecol. Lett..

[27] Leimbacher F., Slusarski W. (1981). Ecology and control of parasite stages in external environment. Ill. An ecological approach to the control of fascioliasis in France. Review of Advances in Parasitology.

[28] Malone J.B. (2005). Biology-based mapping of vector-borne parasites by Geographic Information Systems and Remote Sensing. Parassitologia.

[29] Gillingham E.L., Medlock J.M., Macintyre H., Phalkey R. (2023). Modelling the current and future temperature suitability of the UK for the vector *Hyalomma marginatum* (Acari: Ixodidae). Ticks Tick-Borne Dis..

[30] Nieto P., Malone J.B., Bavia M.E. (2006). Ecological niche modeling for visceral leishmaniasis in the state of Bahia, Brazil, using genetic algorithm for rule-set prediction and growing degree day-water budget analysis. Geospat. Health.

[31] Cuervo P.F., Fantozzi M.C., Di Cataldo S., Cringoli G., Sierra R.M.Y., Rinaldi L. (2013). Analysis of climate and extrinsic incubation of *Dirofilaria immitis* in southern South America. Geospat. Health.

[32] Cuervo P.F., Rinaldi L., Cringoli G. (2015). Modeling the extrinsic incubation of *Dirofilaria immitis* in South America based on monthly and continuous climatic data. Vet. Parasitol..

[33] Salahi-Moghaddam A., Turki H., Yeryan M., Fuentes M.V. (2022). Spatio-temporal Prediction of the Malaria Transmission Risk in Minab District (Hormozgan Province, Southern Iran). Acta Parasitol..

[34] Ewing D.A., Purse B.V., Cobbold C.A., White S.M. (2021). A novel approach for predicting risk of vector-borne disease establishment in marginal temperate environments under climate change: West Nile virus in the UK. J. R. Soc. Interface.

[35] Paz S. (2015). Climate change impacts on West Nile virus transmission in a global context. Philos. Trans. R. Soc. B Biol. Sci..

[36] Beesley N.J., Caminade C., Charlier J., Flynn R.J., Hodgkinson J.E., Martinez-Moreno A., Martinez-Valladares M., Perez J., Rinaldi L., Williams D.J.L. (2018). Fasciola and fasciolosis in ruminants in Europe: Identifying research needs. Transbound. Emerg. Dis..

[37] Beltrame L., Dunne T., Vineer H.R., Walker J.G., Morgan E.R., Vickerman P., McCann C.M., Williams D.J.L., Wagener T. (2018). A mechanistic hydro-epidemiological model of liver fluke risk. J. R. Soc. Interface.

[38] Estrada-Peña A., Ayllón N., de la Fuente J. (2012). Impact of Climate Trends on Tick-Borne Pathogen Transmission. Front. Physiol..

[39] Tjaden N.B., Caminade C., Beierkuhnlein C., Thomas S.M. (2018). Mosquito-Borne Diseases: Advances in Modelling Climate-Change Impacts. Trends Parasitol..

[40] Lafferty K.D. (2009). The ecology of climate change and infectious diseases. Ecology.

[41] Anderson R.P. (2013). A framework for using niche models to estimate impacts of climate change on species distributions. Ann. N. Y. Acad. Sci..

[42] Peterson A.T., Samy A.M. (2016). Geographic potential of disease caused by *Ebola* and *Marburg viruses* in Africa. Acta Trop..

[43] Cheng Y., Tjaden N.B., Jaeschke A., Lühken R., Ziegler U., Thomas S.M., Beierkuhnlein C. (2018). Evaluating the risk for *Usutu virus* circulation in Europe: Comparison of environmental niche models and epidemiological models. Int. J. Health Geogr..

[44] Johnson E.E., Escobar L.E., Zambrana-Torrelio C. (2019). An Ecological Framework for Modeling the Geography of Disease Transmission. Trends Ecol. Evol..

[45] Peterson A.T. (2008). Biogeography of diseases: A framework for analysis. Sci. Nat..

[46] Colwell R.K., Rangel T.F. (2009). Hutchinson’s duality: The once and future niche. Proc. Natl. Acad. Sci. USA.

[47] Soberón J., Osorio-Olvera L., Peterson T. (2017). Diferencias conceptuales entre modelación de nichos y modelación de áreas de distribución. Rev. Mex. Biodivers..

[48] Palomar A.M., Portillo A., Mazuelas D., Roncero L., Arizaga J., Crespo A., Gutiérrez Ó., Márquez F.J., Cuadrado J.F., Eiros J.M. (2016). Molecular analysis of Crimean-Congo hemorrhagic fever virus and Rickettsia in *Hyalomma marginatum* ticks removed from patients (Spain) and birds (Spain and Morocco), 2009–2015. Ticks Tick-Borne Dis..

[49] Sadeghieh T., Waddell L.A., Ng V., Hall A., Sargeant J. (2020). A scoping review of importation and predictive models related to vector-borne diseases, pathogens, reservoirs, or vectors (1999–2016). PLoS ONE.

[50] Lippi C.A., Gaff H.D., White A.L., Ryan S.J. (2021). Scoping review of distribution models for selected *Amblyomma* ticks and rickettsial group pathogens. PeerJ.

[51] Barker J.R., MacIsaac H.J. (2022). Species distribution models applied to mosquitoes: Use, quality assessment, and recommendations for best practice. Ecol. Model..

[52] Kopsco H.L., Smith R.L., Halsey S.J. (2022). A Scoping Review of Species Distribution Modeling Methods for Tick Vectors. Front. Ecol. Evol..

[53] Molina-Guzmán L.P., Gutiérrez-Builes L.A., Ríos-Osorio L.A. (2022). Models of spatial analysis for vector-borne diseases studies: A systematic review. Vet. World.

[54] Moutinho S., Rocha J., Gomes A., Gomes B., Ribeiro A.I. (2022). Spatial Analysis of Mosquito-Borne Diseases in Europe: A Scoping Review. Sustainability.

[55] Peterson A.T., Soberón J., Pearson R.G., Anderson R.P., Martínez-Meyer E., Nakamura M., Araújo M.B. (2011). Ecological niches and geographic distributions. Monographs in Population Biology (Volume 49).

[56] Soberon J., Peterson A.T. (2005). Interpretation of Models of Fundamental Ecological Niches and Species’ Distributional Areas. Biodivers. Inform..

[57] Soberón J. (2007). Grinnellian and Eltonian niches and geographic distributions of species. Ecol. Lett..

[58] Barve N., Barve V., Jiménez-Valverde A., Lira-Noriega A., Maher S.P., Townsend Peterson A., Soberon J., Villalobos F. (2011). The crucial role of the accessible area in ecological niche modeling and species distribution modeling. Ecol. Model..

[59] Owens H.L., Campbell L.P., Dornak L.L., Saupe E.E., Barve N., Soberón J., Ingenloff K., Lira-Noriega A., Hensz C.M., Myers C.E. (2013). Constraints on interpretation of ecological niche models by limited environmental ranges on calibration areas. Ecol. Model..

[60] Sillero N., Arenas-Castro S., Enriquez-Urzelai U., Vale C.G., Sousa-Guedes D., Martínez-Freiría F., Real R., Barbosa A. (2021). Want to model a species niche? A step-by-step guideline on correlative ecological niche modelling. Ecol. Model..

[61] Peterson A.T. (2010). Ecological niche modelling and understanding the geography of disease transmission. Vet. Ital..

[62] Higgins S.I., Larcombe M.J., Beeton N.J., Conradi T., Nottebrock H. (2020). Predictive ability of a process-based versus a correlative species distribution model. Ecol. Evol..

[63] Phillips S.J., Anderson R.P., Schapire R.E. (2006). Maximum entropy modeling of species geographic distributions. Ecol. Model..

[64] Feng X., Park D.S., Walker C., Peterson A.T., Merow C., Papeş M. (2019). A checklist for maximizing reproducibility of ecological niche models. Nat. Ecol. Evol..

[65] Sillero N. (2011). What does ecological modelling model? A proposed classification of ecological niche models based on their under-lying methods. Ecol. Modell..

[66] Peterson A.T. (2014). Mapping Disease Transmission Risk: Geographic and Ecological Contexts.

[67] Andreo V., Rosa J., Ramos K., Salomón O.D. (2022). Ecological characterization of a cutaneous leishmaniasis outbreak through remotely sensed land cover changes. Geospat. Health.

[68] Assefa A., Tibebu A., Bihon A., Dagnachew A., Muktar Y. (2022). Ecological niche modeling predicting the potential distribution of African horse sickness virus from 2020 to 2060. Sci. Rep..

[69] Chaves A., Dolz G., Ibarra-Cerdeña C.N., Núñez G., Ortiz-Malavasi E.E., Bernal-Valle S., Gutiérrez-Espeleta G.A. (2022). Presence and potential distribution of malaria-infected New World primates of Costa Rica. Malar. J..

[70] Celone M., Pecor D.B., Potter A., Richardson A., Dunford J., Pollett S. (2022). An ecological niche model to predict the geographic distribution of *Haemagogus janthinomys*, Dyar, 1921 a yellow fever and *Mayaro virus* vector, in South America. PLoS Negl. Trop. Dis..

[71] Escobar L.E., Craft M.E. (2016). Advances and Limitations of Disease Biogeography Using Ecological Niche Modeling. Front. Microbiol..

[72] Lozier J.D., Aniello P., Hickerson M.J. (2009). Predicting the distribution of Sasquatch in western North America: Anything goes with ecological niche modelling. J. Biogeogr..

[73] Harbach R.E. (2012). Culex pipiens: Species Versus Species Complex–Taxonomic History and Perspective. J. Am. Mosq. Control Assoc..

[74] Tennessen J.A., Ingham V.A., Toé K.H., Guelbéogo W.M., Sagnon N., Kuzma R., Ranson H., Neafsey D.E. (2021). A population genomic unveiling of a new cryptic mosquito taxon within the malaria-transmitting *Anopheles gambiae* complex. Mol. Ecol..

[75] Walton C., Sharpe R.G., Pritchard S.J., Thelwell N.J., Butlin R.K. (1999). Molecular identification of mosquito species. Biol. J. Linn. Soc..

[76] Mas-Coma S., Bargues M. (2009). Populations, hybrids and the systematic concepts of species and subspecies in Chagas disease triatomine vectors inferred from nuclear ribosomal and mitochondrial DNA. Acta Trop..

[77] Cruz D.D., Arellano E. (2022). Molecular data confirm *Triatoma pallidipennis* Stål, 1872 (Hemiptera: Reduviidae: Triatominae) as a novel cryptic species complex. Acta Trop..

[78] Abad-Franch F., Monteiro F.A., Pavan M.G., Patterson J.S., Bargues M.D., Zuriaga M.Á., Aguilar M., Beard C.B., Mas-Coma S., Miles M.A. (2021). Under pressure: Phenotypic divergence and convergence associated with microhabitat adaptations in Triatominae. Parasites Vectors.

[79] Gutierrez M.A.C., Lopez R.O.H., Ramos A.T., Vélez I.D., Gomez R.V., Arrivillaga-Henríquez J., Uribe S. (2021). DNA barcoding of *Lutzomyia longipalpis* species complex (Diptera: Psychodidae), suggests the existence of 8 candidate species. Acta Trop..

[80] Calzolari M., Desiato R., Albieri A., Bellavia V., Bertola M., Bonilauri P., Callegari E., Canziani S., Lelli D., Mosca A. (2021). Mosquitoes of the Maculipennis complex in Northern Italy. Sci. Rep..

[81] Cuervo P.F., Artigas P., Mas-Coma S., Bargues M.D. (2022). West Nile virus in Spain: Forecasting the geographical distribution of risky areas with an ecological niche modelling approach. Transbound. Emerg. Dis..

[82] Hernández C., Alvarado M., Salgado-Roa F.C., Ballesteros N., Rueda-M N., Oliveira J., Alevi K.C.C., da Rosa J.A., Urbano P., Salazar C. (2022). Phylogenetic relationships and evolutionary patterns of the genus *Psammolestes* Bergroth, 1911 (Hemiptera: Reduviidae: Triatominae). BMC Ecol. Evol..

[83] de Beer C.J., Dicko A.H., Ntshangase J., Moyaba P., Taioe M.O., Mulandane F.C., Neves L., Mdluli S., Guerrini L., Bouyer J. (2021). A distribution model for *Glossina brevipalpis* and *Glossina austeni* in Southern Mozambique, Eswatini and South Africa for enhanced area-wide integrated pest management approaches. PLoS Negl. Trop. Dis..

[84] Fonseca E.D.S., Guimarães R.B., Prestes-Carneiro L.E., Tolezano J.E., Rodgers M.D.S.M., Avery R.H., Malone J.B. (2021). Predicted distribution of sand fly (Diptera: Psychodidae) species involved in the transmission of Leishmaniasis in São Paulo state, Brazil, utilizing maximum entropy ecological niche modeling. Pathog. Glob. Health.

[85] Rodgers M.D.S.M., Fonseca E., Nieto P.D.M., Malone J.B., Luvall J.C., McCarroll J.C., Avery R.H., Bavia M.E., Guimaraes R., Wen X. (2022). Use of soil moisture active passive satellite data and WorldClim 2.0 data to predict the potential distribution of visceral leishmaniasis and its vector *Lutzomyia longipalpis* in Sao Paulo and Bahia states, Brazil. Geospat. Health.

[86] Fetene E., Teka G., Dejene H., Mandefro D., Teshome T., Temesgen D., Negussie H., Mulatu T., Jaleta M.B., Leta S. (2022). Modeling the spatial distribution of *Culicoides* species (Diptera: Ceratopogonidae) as vectors of animal diseases in Ethiopia. Sci. Rep..

[87] Flores-López C.A., Moo-Llanes D.A., Romero-Figueroa G., Guevara-Carrizales A., López-Ordoñez T., Casas-Martínez M., Samy A.M. (2022). Potential distributions of the parasite *Trypanosoma cruzi* and its vector *Dipetalogaster maxima* highlight areas at risk of Chagas disease transmission in Baja California Sur, Mexico, under climate change. Med. Vet. Entomol..

[88] Gachoki S., Groen T., Vrieling A., Okal M., Skidmore A., Masiga D. (2021). Satellite-based modelling of potential tsetse (*Glossina pallidipes*) breeding and foraging sites using teneral and non-teneral fly occurrence data. Parasites Vectors.

[89] McBride S.E., Lieberthal B.A., Buttke D.E., Cronk B.D., De Urioste-Stone S.M., Goodman L.B., Guarnieri L.D., Rounsville T.F., Gardner A.M. (2023). Patterns and Ecological Mechanisms of Tick-Borne Disease Exposure Risk in Acadia National Park, Mount Desert Island, Maine, United States. J. Med. Entomol..

[90] Moua Y., Kotchi S., Ludwig A., Brazeau S. (2021). Mapping the Habitat Suitability of West Nile Virus Vectors in Southern Quebec and Eastern Ontario, Canada, with Species Distribution Modeling and Satellite Earth Observation Data. Remote Sens..

[91] Nurjanah S., Atmowidi T., Hadi U.K., Solihin D.D., Priawandiputra W., Santoso B. (2022). Distribution modelling of *Aedes aegypti* in three dengue-endemic areas in Sumatera, Indonesia. Trop. Biomed..

[92] Omar K., Thabet H., TagEldin R., Asadu C., Chukwuekezie O., Ochu J., Dogunro F., Nwangwu U., Onwude O., Ezihe E. (2021). Ecological niche modeling for predicting the potential geographical distribution of *Aedes* species (Diptera: Culicidae): A case study of Enugu State, Nigeria. Parasite Epidemiol. Control.

[93] Springer A., Lindau A., Probst J., Drehmann M., Fachet K., Thoma D., Vineer H.R., Noll M., Dobler G., Mackenstedt U. (2022). Update and prognosis of Dermacentor distribution in Germany: Nationwide occurrence of *Dermacentor reticulatus*. Front. Vet. Sci..

[94] Tagwireyi P., Ndebele M., Chikurunhe W. (2022). Climate change diminishes the potential habitat of the bont tick (*Amblyomma hebraeum*): Evidence from Mashonaland Central Province, Zimbabwe. Parasites Vectors.

[95] Porter W.T., Barrand Z.A., Wachara J., DaVall K., Mihaljevic J.R., Pearson T., Salkeld D.J., Nieto N.C. (2021). Predicting the current and future distribution of the western black-legged tick, Ixodes pacificus, across the Western US using citizen science collections. PLoS ONE.

[96] Thameur B.H., Soufiène S., Ammar H.H., Hammami S. (2021). Spatial distribution and habitat selection of culicoides imicola: The potential vector of bluetongue virus in Tunisia. Onderstepoort J. Vet. Res..

[97] Cuervo P.F., Flores F.S., Venzal J.M., Nava S. (2021). Niche divergence among closely related taxa provides insight on evolutionary patterns of ticks. J. Biogeogr..

[98] Rengifo-Correa L., González-Salazar C., Stephens C.R. (2023). Disentangling the contributions of biotic and abiotic predictors in the niche and the species distribution model of *Trypanosoma cruzi*, etiological agent of Chagas disease. Acta Trop..

[99] Furlong M., Adamu A., Hickson R.I., Horwood P., Golchin M., Hoskins A., Russell T. (2022). Estimating the Distribution of Japanese Encephalitis Vectors in Australia Using Ecological Niche Modelling. Trop. Med. Infect. Dis..

[100] Adeleke E.D., Shittu R.A., Beierkuhnlein C., Thomas S.M. (2022). High Wind Speed Prevents the Establishment of the Disease Vector Mosquito *Aedes albopictus* in Its Climatic Niche in Europe. Front. Environ. Sci..

[101] Al-Obaidi M.J., Ali H.B. (2021). Effect of Climate Change on the Distribution of Zoonotic Cutaneous Leishmaniasis in Iraq. J. Phys. Conf. Ser..

[102] Campbell L., Burkett-Cadena N., Miqueli E., Unlu I., Sloyer K., Medina J., Vasquez C., Petrie W., Reeves L. (2021). Potential Distribution of *Aedes* (*Ochlerotatus*) *scapularis* (Diptera: Culicidae): A Vector Mosquito New to the Florida Peninsula. Insects.

[103] Gorris M.E., Bartlow A.W., Temple S.D., Romero-Alvarez D., Shutt D.P., Fair J.M., Kaufeld K.A., Del Valle S.Y., Manore C.A. (2021). Updated distribution maps of predominant *Culex mosquitoes* across the Americas. Parasites Vectors.

[104] Holeva-Eklund W.M., Young S.J., Will J., Busser N., Townsend J., Hepp C.M. (2022). Species distribution modeling of *Aedes aegypti* in Maricopa County, Arizona from 2014 to 2020. Front. Environ. Sci..

[105] Hussain S.S.A., Dhiman R.C. (2022). Distribution Expansion of Dengue Vectors and Climate Change in India. Geohealth.

[106] Ocampo C.B., Guzmán-Rodríguez L., Moreno M., Castro M.D.M., Valderrama-Ardila C., Alexander N. (2021). Integration of phlebotomine ecological niche modelling, and mapping of cutaneous leishmaniasis surveillance data, to identify areas at risk of under-estimation. Acta Trop..

[107] Rhodes C.G., Loaiza J.R., Romero L.M., Alvarado J.M.G., Delgado G., Salas O.R., Rojas M.R., Aguilar-Avendaño C., Maynes E., Cordero J.A.V. (2022). *Anopheles albimanus* (Diptera: Culicidae) Ensemble Distribution Modeling: Applications for Malaria Elimination. Insects.

[108] Valderrama L., Ayala S., Reyes C., González C.R. (2021). Modeling the Potential Distribution of the Malaria Vector *Anopheles* (*Ano.*) *pseudopunctipennis* Theobald (Diptera: Culicidae) in Arid Regions of Northern Chile. Front. Public Health.

[109] Sánchez-Fernández D., Lobo J.M., Hernández-Manrique O.L. (2011). Species distribution models that do not incorporate global data misrepresent potential distributions: A case study using Iberian diving beetles. Divers. Distrib..

[110] Raes N. (2012). Partial versus Full Species Distribution Models. Nat. Conserv..

[111] Chevalier M., Zarzo-Arias A., Guélat J., Mateo R.G., Guisan A. (2022). Accounting for niche truncation to improve spatial and temporal predictions of species distributions. Front. Ecol. Evol..

[112] Li Y.-P., Gao X., An Q., Sun Z., Wang H.-B. (2022). Ecological niche modeling based on ensemble algorithms to predicting current and future potential distribution of African swine fever virus in China. Sci. Rep..

[113] Amdouni J., Conte A., Ippoliti C., Candeloro L., Tora S., Sghaier S., Ben Hassine T., Fakhfekh E.A., Savini G., Hammami S. (2022). *Culex pipiens* distribution in Tunisia: Identification of suitable areas through Random Forest and MaxEnt approaches. Vet. Med. Sci..

[114] Andreo V., Cuervo P.F., Porcasi X., Lopez L., Guzman C., Scavuzzo C.M. (2021). Towards a workflow for operational mapping of Aedes aegypti at urban scale based on remote sensing. Remote Sens. Appl. Soc. Environ..

[115] Hahn M.B., Feirer S., Monaghan A.J., Lane R.S., Eisen R.J., Padgett K.A., Kelly M. (2021). Modeling future climate suitability for the western blacklegged tick, *Ixodes pacificus*, in California with an emphasis on land access and ownership. Ticks Tick-Borne Dis..

[116] Lippi C.A., Gaff H.D., White A.L., John H.K.S., Richards A.L., Ryan S.J. (2021). Exploring the Niche of *Rickettsia montanensis* (*Rickettsiales*: *Rickettsiaceae*) Infection of the American Dog Tick (Acari: Ixodidae), Using Multiple Species Distribution Model Approaches. J. Med. Entomol..

[117] Moo-Llanes D., López-Ordóñez T., Torres-Monzón J., Mosso-González C., Casas-Martínez M., Samy A. (2021). Assessing the Potential Distributions of the Invasive Mosquito Vector *Aedes albopictus* and Its Natural *Wolbachia* Infections in México. Insects.

[118] Chevalier M., Broennimann O., Cornuault J., Guisan A. (2021). Data integration methods to account for spatial niche truncation effects in regional projections of species distribution. Ecol. Appl..

[119] Tjaden N., Cheng Y., Beierkuhnlein C., Thomas S. (2021). Chikungunya Beyond the Tropics: Where and When Do We Expect Disease Transmission in Europe?. Viruses.

[120] Tjaden N.B., Suk J.E., Fischer D., Thomas S.M., Beierkuhnlein C., Semenza J.C. (2017). Modelling the effects of global climate change on Chikungunya transmission in the 21st century. Sci. Rep..

[121] Ochida N., Mangeas M., Dupont-Rouzeyrol M., Dutheil C., Forfait C., Peltier A., Descloux E., Menkes C. (2022). Modeling present and future climate risk of dengue outbreak, a case study in New Caledonia. Environ. Health.

[122] Cunze S., Kochmann J., Klimpel S. (2020). Global occurrence data improve potential distribution models for *Aedes japonicus japonicus* in non-native regions. Pest Manag. Sci..

[123] Yañez-Arenas C., Rioja-Nieto R., A Martín G., Dzul-Manzanilla F., Chiappa-Carrara X., Buenfil-Ávila A., Manrique-Saide P., Morales F.C., Díaz-Quiñónez J.A., Pérez-Rentería C. (2018). Characterizing environmental suitability of *Aedes albopictus* (Diptera: Culicidae) in Mexico based on regional and global niche models. J. Med. Entomol..

[124] Echeverry-Cárdenas E., López-Castañeda C., Carvajal-Castro J.D., Aguirre-Obando O.A. (2021). Potential geographic distribution of the tiger mosquito *Aedes albopictus* (Skuse, 1894) (Diptera: Culicidae) in current and future conditions for Colombia. PLoS Negl. Trop. Dis..

[125] Titeux N., Maes D., Van Daele T., Onkelinx T., Heikkinen R.K., Romo H., García-Barros E., Munguira M.L., Thuiller W., Van Swaay C.A.M. (2017). The need for large-scale distribution data to estimate regional changes in species richness under future climate change. Divers. Distrib..

[126] Carretero M.A., Sillero N. (2016). Evaluating how species niche modelling is affected by partial distributions with an empirical case. Acta Oecol..

[127] Phillips S.J., Elith J. (2013). On estimating probability of presence from use–availability or presence–background data. Ecology.

[128] Merow C., Smith M.J., Silander J.A. (2013). A practical guide to MaxEnt for modeling species’ distributions: What it does, and why inputs and settings matter. Ecography.

[129] Anderson R.P., Raza A. (2010). The effect of the extent of the study region on GIS models of species geographic distributions and estimates of niche evolution: Preliminary tests with montane rodents (genus *Nephelomys*) in Venezuela. J. Biogeogr..

[130] Nekola J.C., Divíšek J., Horsák M. (2022). The nature of dispersal barriers and their impact on regional species pool richness and turnover. Glob. Ecol. Biogeogr..

[131] Pang S.E.H., Zeng Y., Alban J.D.T., Webb E.L. (2022). Occurrence–habitat mismatching and niche truncation when modelling distributions affected by anthropogenic range contractions. Divers. Distrib..

[132] Machado-Stredel F., Cobos M.E., Peterson A.T. (2021). A simulation-based method for selecting calibration areas for ecological niche models and species distribution models. Front. Biogeogr..

[133] Escalante T. (2009). Un ensayo sobre regionalización biogeográfica. Rev. Mex. Biodivers..

[134] Morrone J.J. (2018). The spectre of biogeographical regionalization. J. Biogeogr..

[135] Sierra-Morales P., Rojas-Soto O., Ríos-Muñoz C.A., Ochoa-Ochoa L.M., Flores-Rodríguez P., Almazán-Núñez R.C. (2021). Climate change projections suggest severe decreases in the geographic ranges of bird species restricted to Mexican humid mountain forests. Glob. Ecol. Conserv..

[136] Ramsey J.M., Peterson A.T., Carmona-Castro O., Moo-Llanes D.A., Nakazawa Y., Butrick M., Tun-Ku E., De La Cruz-Félix K., Ibarra-Cerdeña C.N. (2015). Atlas of Mexican Triatominae (Reduviidae: Hemiptera) and vector transmission of Chagas disease. Mem. Inst. Oswaldo Cruz.

[137] Soberón J.M. (2010). Niche and area of distribution modeling: A population ecology perspective. Ecography.

[138] Bystriakova N., Peregrym M., Erkens R.H., Bezsmertna O., Schneider H. (2012). Sampling bias in geographic and environmental space and its effect on the predictive power of species distribution models. Syst. Biodivers..

[139] Hughes A.C., Orr M.C., Ma K., Costello M.J., Waller J., Provoost P., Yang Q., Zhu C., Qiao H. (2021). Sampling biases shape our view of the natural world. Ecography.

[140] Rocha-Ortega M., Rodriguez P., Córdoba-Aguilar A. (2021). Geographical, temporal and taxonomic biases in insect GBIF data on biodiversity and extinction. Ecol. Entomol..

[141] Phillips S.J., Dudík M., Elith J., Graham C.H., Lehmann A., Leathwick J., Ferrier S. (2009). Sample selection bias and presence-only distribution models: Implications for background and pseudo-absence data. Ecol. Appl..

[142] Laporta G.Z., Potter A.M., Oliveira J.F.A., Bourke B.P., Pecor D.B., Linton Y.-M. (2023). Global Distribution of *Aedes aegypti* and *Aedes albopictus* in a Climate Change Scenario of Regional Rivalry. Insects.

[143] Aiello-Lammens M.E., Boria R.A., Radosavljevic A., Vilela B., Anderson R.P. (2015). spThin: An R package for spatial thinning of species occurrence records for use in ecological niche models. Ecography.

[144] Castellanos A.A., Huntley J.W., Voelker G., Lawing A.M. (2019). Environmental filtering improves ecological niche models across multiple scales. Methods Ecol. Evol..

[145] Kramer-Schadt S., Niedballa J., Pilgrim J.D., Schröder B., Lindenborn J., Reinfelder V., Stillfried M., Heckmann I., Scharf A.K., Augeri D.M. (2013). The importance of correcting for sampling bias in MaxEnt species distribution models. Divers. Distrib..

[146] Boria R.A., Olson L.E., Goodman S.M., Anderson R.P. (2014). Spatial filtering to reduce sampling bias can improve the performance of ecological niche models. Ecol. Model..

[147] Varela S., Anderson R.P., García-Valdés R., Fernández González F. (2014). Environmental filters reduce the effects of sampling bias and improve predictions of ecological niche models. Ecography.

[148] Cosentino F., Maiorano L. (2021). Is geographic sampling bias representative of environmental space?. Ecol. Inform..

[149] Barber R.A., Ball S.G., Morris R.K.A., Gilbert F. (2022). Target-group backgrounds prove effective at correcting sampling bias in Maxent models. Divers. Distrib..

[150] Vollering J., Halvorsen R., Auestad I., Rydgren K. (2019). Bunching up the background betters bias in species distribution models. Ecography.

[151] De Oliveira G., Rangel T.F., Lima-Ribeiro M.S., Terribile L.C., Diniz-Filho J.A.F. (2014). Evaluating, partitioning, and mapping the spatial autocorrelation component in ecological niche modeling: A new approach based on environmentally equidistant records. Ecography.

[152] Peterson A.T., Cobos M.E., Jiménez-García D. (2018). Major challenges for correlational ecological niche model projections to future climate conditions. Ann. N. Y. Acad. Sci..

[153] Warren D.L., Seifert S.N. (2011). Ecological niche modeling in Maxent: The importance of model complexity and the performance of model selection criteria. Ecol. Appl..

[154] Peterson A.T., Nakazawa Y. (2008). Environmental data sets matter in ecological niche modelling: An example with *Solenopsis invicta* and *Solenopsis richteri*. Glob. Ecol. Biogeogr..

[155] Cobos M.E., Peterson A.T., Osorio-Olvera L., Jiménez-García D. (2019). An exhaustive analysis of heuristic methods for variable selection in ecological niche modeling and species distribution modeling. Ecol. Inform..

[156] Zurell D., Franklin J., König C., Bouchet P.J., Dormann C.F., Elith J., Fandos G., Feng X., Guillera-Arroita G., Guisan A. (2020). A standard protocol for reporting species distribution models. Ecography.

[157] Muscarella R., Galante P.J., Soley-Guardia M., Boria R.A., Kass J.M., Uriarte M., Anderson R.P. (2014). ENMeval: An R package for conducting spatially independent evaluations and estimating optimal model complexity for Maxentecological niche models. Methods Ecol. Evol..

[158] Vignali S., Barras A.G., Arlettaz R., Braunisch V. (2020). *SDMtune*: An R package to tune and evaluate species distribution models. Ecol. Evol..

[159] Radosavljevic A., Anderson R.P. (2014). Making better Maxent models of species distributions: Complexity, overfitting and evaluation. J. Biogeogr..

[160] Kass J.M., Muscarella R., Galante P.J., Bohl C.L., Pinilla-Buitrago G.E., Boria R.A., Soley-Guardia M., Anderson R.P. (2021). ENMeval 2.0: Redesigned for customizable and reproducible modeling of species’ niches and distributions. Methods Ecol. Evol..

[161] Kass J.M., Vilela B., Aiello-Lammens M.E., Muscarella R., Merow C., Anderson R.P. (2018). Wallace: A flexible platform for reproducible modeling of species niches and distributions built for community expansion. Methods Ecol. Evol..

[162] Kass J.M., Pinilla-Buitrago G.E., Paz A., Johnson B.A., Grisales-Betancur V., Meenan S.I., Attali D., Broennimann O., Galante P.J., Maitner B.S. (2023). *wallace* 2: A shiny app for modeling species niches and distributions redesigned to facilitate expansion via module contributions. Ecography.

[163] Cobos M.E., Townsend Peterson A., Barve N., Osorio-Olvera L. (2019). kuenm: An R package for detailed development of ecological niche models using Maxent. PeerJ.

[164] Wiens J.A., Stralberg D., Jongsomjit D., Howell C.A., Snyder M.A. (2009). Niches, models, and climate change: Assessing the assumptions and uncertainties. Proc. Natl. Acad. Sci. USA.

[165] Pearson R.G., Thuiller W., Araújo M.B., Martínez-Meyer E., Brotons L., McClean C., Miles L., Segurado P., Dawson T., Lees D.C. (2006). Model-based uncertainty in species range prediction. J. Biogeogr..

[166] Alkishe A., Cobos M.E., Peterson A.T., Samy A.M. (2020). Recognizing sources of uncertainty in disease vector ecological niche models: An example with the tick *Rhipicephalus sanguineus* sensu lato. Perspect. Ecol. Conserv..

[167] Qiao H., Feng X., Escobar L.E., Peterson A.T., Soberón J., Zhu G., Papeş M. (2019). An evaluation of transferability of ecological niche models. Ecography.

[168] Simoes M., Romero-Alvarez D., Nuñez-Penichet C., Jiménez L., Cobos M.E. (2020). General Theory and Good Practices in Ecological Niche Modeling: A Basic Guide. Biodivers. Inform..

[169] Elith J., Kearney M., Phillips S. (2010). The art of modelling range-shifting species. Methods Ecol. Evol..

[170] Witmer F.D.W., Nawrocki T.W., Hahn M. (2022). Modeling Geographic Uncertainty in Current and Future Habitat for Potential Populations of *Ixodes pacificus* (Acari: Ixodidae) in Alaska. J. Med. Entomol..

[171] Yates K.L., Bouchet P.J., Caley M.J., Mengersen K., Randin C.F., Parnell S., Fielding A.H., Bamford A.J., Ban S., Barbosa A.M. (2018). Outstanding Challenges in the Transferability of Ecological Models. Trends Ecol. Evol..

[172] Alkhamis M.A., Fountain-Jones N.M., Aguilar-Vega C., Sánchez-Vizcaíno J.M. (2021). Environment, vector, or host? Using machine learning to untangle the mechanisms driving arbovirus outbreaks. Ecol. Appl..

[173] Peterson A.T. (2006). Ecologic Niche Modeling and Spatial Patterns of Disease Transmission. Emerg. Infect. Dis..

[174] Lockwood J.L., Hoopes M.F., Marchetti M.P. (2013). Invasion Ecology.

[175] Wilson J.R., Dormontt E.E., Prentis P.J., Lowe A.J., Richardson D.M. (2009). Something in the way you move: Dispersal pathways affect invasion success. Trends Ecol. Evol..

[176] Crowl T.A., Crist T.O., Parmenter R.R., Belovsky G., Lugo A.E. (2008). The spread of invasive species and infectious disease as drivers of ecosystem change. Front. Ecol. Environ..

[177] Jimenezvalverde A., Peterson A.T., Soberon J., Overton J.M., Aragón P., Lobo J.M. (2011). Use of niche models in invasive species risk assessments. Biol. Invasions.

[178] Castaño-Quintero S., Escobar-Luján J., Osorio-Olvera L., Peterson A.T., Chiappa-Carrara X., Martínez-Meyer E., Yañez-Arenas C. (2020). Supraspecific units in correlative niche modeling improves the prediction of geographic potential of biological invasions. PeerJ.

[179] Sanderson B.M., Knutti R., Caldwell P. (2015). A Representative Democracy to Reduce Interdependency in a Multimodel Ensemble. J. Clim..

[180] Chen I.-C., Hill J.K., Ohlemüller R., Roy D.B., Thomas C.D. (2011). Rapid Range Shifts of Species Associated with High Levels of Climate Warming. Science.

[181] Pili A.N., Tingley R., Sy E.Y., Diesmos M.L.L., Diesmos A.C. (2020). Niche shifts and environmental non-equilibrium undermine the usefulness of ecological niche models for invasion risk assessments. Sci. Rep..

[182] Chuang A., Peterson C.R. (2016). Expanding population edges: Theories, traits, and trade-offs. Glob. Chang. Biol..

[183] Arenas M., Ray N., Currat M., Excoffier L. (2012). Consequences of Range Contractions and Range Shifts on Molecular Diversity. Mol. Biol. Evol..

[184] Cunze S., Glock G., Kochmann J., Klimpel S. (2022). Ticks on the move—Climate change-induced range shifts of three tick species in Europe: Current and future habitat suitability for *Ixodes ricinus* in comparison with *Dermacentor reticulatus* and *Dermacentor marginatus*. Parasitol. Res..

[185] Flenniken J.M., Tuten H.C., Vineer H.R., Phillips V.C., Stone C.M., Allan B.F. (2022). Environmental Drivers of Gulf Coast Tick (Acari: Ixodidae) Range Expansion in the United States. J. Med. Entomol..

[186] Alkishe A., Raghavan R.K., Peterson A.T. (2021). Likely Geographic Distributional Shifts among Medically Important Tick Species and Tick-Associated Diseases under Climate Change in North America: A Review. Insects.

[187] Steger J., Schneider A., Brandl R., Hotes S. (2020). Effects of projected climate change on the distribution of *Mantis religiosa* suggest expansion followed by contraction. Web Ecol..

[188] McIntyre S., Rangel E.F., Ready P.D., Carvalho B.M. (2017). Species-specific ecological niche modelling predicts different range contractions for *Lutzomyia intermedia* and a related vector of *Leishmania braziliensis* following climate change in South America. Parasites Vectors.

[189] Wiens J.J. (2007). Species Delimitation: New Approaches for Discovering Diversity. Syst. Biol..

[190] Alvarado-Serrano D.F., Knowles L.L. (2014). Ecological niche models in phylogeographic studies: Applications, advances and precautions. Mol. Ecol. Resour..

[191] Wiens J.J., Graham C.H. (2005). Niche Conservatism: Integrating Evolution, Ecology, and Conservation Biology. Annu. Rev. Ecol. Evol. Syst..

[192] Foley D.H., Rueda L.M., Peterson A.T., Wilkerson R.C. (2008). Potential Distribution of Two Species in the Medically Important *Anopheles minimus* Complex (Diptera: Culicidae). J. Med. Entomol..

[193] Collart F., Hedenäs L., Broennimann O., Guisan A., Vanderpoorten A. (2021). Intraspecific differentiation: Implications for niche and distribution modelling. J. Biogeogr..

